# Application of Size Exclusion Chromatography with Multiangle Light Scattering in the Analytical Development of a Preclinical Stage Gene Therapy Program

**DOI:** 10.1089/hum.2022.218

**Published:** 2023-04-17

**Authors:** Bryan Troxell, I-Wei Tsai, Kinjal Shah, Christopher I. Knuckles, Sarah Shelton, Kate Lindsey, Selene M. Barbosa Cardenas, Taylor Roberts

**Affiliations:** ^1^ Analytical Development and Quality Control, StrideBio, Research Triangle Park, North Carolina, USA.; ^2^ AjaxBio, LLC, Holly Springs, North Carolina, USA.

**Keywords:** SEC-MALS, ddPCR, gene therapy, SV-AUC, cryo-EM, AAV

## Abstract

To provide safe recombinant adeno-associated viruses (rAAV) to patients, scalable manufacturing processes are required. However, these processes may introduce impurities that impact the performance and quality of the final drug product. Empty rAAV capsids are product-related impurities. Regulatory guidance requires that accurate analytical methods be implemented early in product development to measure the level of empty capsids. A process confirmation vector, produced from 200 L production, was used to develop and optimize a size exclusion chromatography with UV and multiangle light scattering (SEC-MALS) method. Vector produced from a 500 L production was used to assess the full-to-empty ratio using the following analytical methods: sedimentation velocity analytical ultracentrifugation (SV-AUC), droplet digital PCR (ddPCR) with capsid enzyme-linked immunosorbent assay (ELISA), bulk absorbance at 260/280 nm, cryogenic electron microscopy, and SEC-MALS. This test article was used for a 30-day, non-Good Laboratory Practices animal study that assessed biodistribution of the product (STRX-330). SEC-MALS outperformed the other methods and correlated well with SV-AUC values of full-to-empty particles. In addition, SEC-MALS agreed with ddPCR and ELISA measurements for vector genomes/mL and capsid particles/mL, respectively. SEC-MALS was linear, accurate, and precise while achieving chromatography quality control (QC) recommendations. Compared to other stability-indicating assays, SEC-MALS performed similarly to ddPCR, capsid ELISA, and infectivity assays in accelerated stress studies. In response to alkaline, but not acidic stress, SEC-MALS indicated distinct changes in the DNA content of the monomer Adeno-associated viruses (AAV) peak for STRX-330, which was supported by ddPCR data. Conversely, acidic treatment resulted in more aggregated vector, but did not impact the DNA content. This work indicates that SEC-MALS is a valuable analytical tool in the analytical development and QC testing of AAV. In addition, this work suggests that SEC-MALS can provide fundamental understanding of AAV in response to environmental stress. This may impact steps of the manufacturing process to minimize conditions that reduce performance.

## INTRODUCTION

Adeno-associated viruses (AAV) are promising vehicles for the treatment and potential cure of numerous human genetic diseases. These viruses are nonpathogenic, exhibit lower immunogenicity than most viruses, and are capable of transducing a variety of cell types.^1,2^ In addition, a viral vector can package ∼4.7 kb of DNA, be produced at scale to meet clinical trial demands with evolved capsids and tissue-specific cassette design that allow for targeted delivery and expression.^3–6^ The icosahedral AAV capsid is composed of viral proteins (VP) VP1, VP2, and VP3 in an ∼1:1:10 ratio.^7–10^

Recent evidence suggests that the VP ratios comprising an AAV capsid population may be more divergent and variable than earlier data indicated.^11^ The design and directed evolution of VPs provide a unique platform to test the impact of amino acid changes on the immune evasion and tissue tropism of AAV. There will likely be a continued need to improve the manufacturability and *in vivo* efficacy as more genetic indications are targeted for treatment.

Using the search term “adeno associated virus,” there are >100 clinical trials ongoing in the United States that utilize recombinant AAV (rAAV) to deliver genetic cargo to correct genetic diseases (ClinicalTrials.gov). As technology improves and demand increases, the number of approved rAAV gene therapies will likely increase dramatically. Manufacturing of rAAV typically relies on the following genetic components provided *in trans*: *Rep* and *Cap* genes, target DNA flanked by inverted terminal repeats (ITRs), and adenovirus helper genes. These are located on separate DNA molecules to minimize the packaging of off-target DNA sequences. Despite these efforts, residual host and plasmid DNA, partially packaged target DNA, and empty capsids are often purified with rAAV capsids that contain the therapeutic gene of interest.^12–14^

These product-related impurities may cause unwanted results during administration to humans; however, the contribution of empty capsids toward *in vivo* efficacy is not clear. Animal studies with AAV8 demonstrate a reduced efficacy when partially-packaged or empty AAV capsids were present.^12^ Alternatively, other studies have shown that these impurities may act as decoys for the host immune system, thereby improving full vector efficacy.^15,16^ Regardless of the *in vivo* impact, it is clear that for a gene therapy program to advance, analytical capabilities must be able to quantify the levels of empty and full capsids in manufactured AAV.

Previous studies have demonstrated the capability of analytical methods to quantify full, partial, and empty capsids.^17–22^ Both cryogenic electron microscopy (cryo-EM) and sedimentation velocity analytical ultracentrifugation (SV-AUC) have been used to gain fundamental knowledge and to quantify full and empty particles of AAV.^18,23–27^ Mass photometry as well as orbitrap-based charge-detection mass spectrometry have demonstrated utility for quantification of full, partial, and empty capsids.^28,29^ In addition, size exclusion chromatography with UV and multiangle light scattering (SEC-MALS) has been applied to quantify numerous critical quality attributes (CQAs) of AAV.^30,31^ Additional approaches that quantify the nucleic acid content and capsid concentrations have been used to obtain a ratio of genome to capsid content. The absorbance profile of purified rAAV vectors without separation by chromatography has also been used to quantify DNA and protein content, thereby obtaining a full-to-empty ratio.^17^

Arrhythmogenic right ventricular cardiomyopathy (ARVC) is a cardiac condition that is caused by several factors.^32^ Several genetic loci have been associated with manifestation of ARVC,^33^ which suggests that gene therapy may be a viable approach to treatment. To this end, a capsid variant derived from AAV9 was isolated from directed evolution studies, named STRV84, and used for program advancement. In addition, a DNA cassette with tissue-specific regulatory control of a gene believed to improve the ARVC condition was developed. This vector, named STRX-330, was produced within StrideBio as part of preclinical studies. As part of the program advancement, analytical methods were developed. Because full-to-empty quantification is a known CQA for gene therapies, multiple approaches to assess this CQA were performed.

Specifically, SV-AUC, cryo-EM, SEC-MALS, droplet digital PCR (ddPCR)/enzyme-linked immunosorbent assay (ELISA), and bulk absorbance measurements (via the Stunner instrument from Unchained Labs) were used to quantify full and empty particles. A test article from a 200 L process confirmation production was used to develop and optimize a SEC-MALS method. For comparison of all methods, material from a 500 L production was used. This material was also used in a non-Good Laboratory Practices (GLP) animal study for a gene therapy program. Of the tested methods, SEC-MALS displayed the strongest agreement with SV-AUC and required minimal development and optimization. In addition, the concentration of vector genomes (VG) obtained by SEC-MALS was in agreement with DNase-resistant ddPCR and data from the Stunner instrument. Because of the impact of dosing on preclinical studies, the use of multiple methods to quantify VG is beneficial.

Furthermore, forced degradation studies with pH conditions demonstrated that SEC-MALS was similar to ddPCR, capsid ELISA, and a cell-based assay (50% tissue culture infectivity [TCID_50_]) in the ability to measure product instability. The data support that SEC-MALS is a robust analytical technique for advancement of gene therapy programs.

## MATERIALS AND METHODS

### Reagents, chemicals, and supplies

The HeLaRC32 cell line (catalog CRL-2972) and Adenovirus serotype 5 (catalog VR-1516) were purchased from American Type Culture Collection (Manassas, VA). Dulbecco's phosphate-buffered saline (DPBS) and pluronic F-68 were purchased from Gibco (Grand Island, NY). Ultra-pure 10% sodium dodecyl sulfate and Trizma were purchased from Invitrogen (Waltham, MA). Fetal bovine serum (FBS) was purchased from HyClone (Logan, UT). Tween 20, EDTA, 100% ethanol, NaOH, Accelagen, Turbonuclease, and Maxisorp nunc plates were purchased from Fisher Scientific (Waltham, MA). HCl was purchased from Honeywell Fluka (Charlotte, NC). Proteinase K, deoxycholic acid, Blocker Casein in phosphate-buffered saline (PBS), POROS CaptureSelect AAVX affinity resin, CaptureSelect Biotin AAV9, CaptureSelect horseradish peroxidase (HRP) AAV9, 3,3′5,5′-tetramethylbenzidine (TMB) substrate, and sulfuric acid were purchased from Thermo Scientific (Waltham, MA).

Dulbecco's modified Eagle's medium (DMEM), 4-(2-hydroxyethyl)-1-piperazineethanesulfonic acid (HEPES), and 10 × PBS were purchased from Corning (Corning, NY). Liquid chromatography-mass spectrometry grade water and streptavidin were purchased from Pierce (Waltham, MA). Bovine serum albumin (BSA) was purchased from USP (Rockville, MD). 10 × PBS Tween (1%) was purchased from BostonBio Products (Milford, MA). Automated droplet generation cartridges, ddPCR supermix for probes, ddPCR droplet reader oil, pipet tips for automated droplet generator, automated droplet generation oil for probes, ddPCR plate seals, and PCR plates were purchased from Bio-Rad (Carlsbad, CA).

### STRX-330 production

The STRX-330 material used here was produced using a proprietary cell line and process. In brief, a HEK cell suspension of the listed volumes was transfected with three plasmids containing a plasmid with the gene of interest for the treatment of ARVC flanked by ITR, a plasmid containing the AAV2 *Rep* and STRV84 *Cap*, and a proprietary helper virus plasmid. The gene cassette present on the ITR plasmid was designed to minimize off-target DNA packaging (*i.e.,* residual and overpackaging). rAAV was produced and the cell culture was lysed using a commercially available detergent, treated with turbo nuclease (Accelagen), and then incubated at 37°C for several hours. Clarified and filtered lysate material was loaded onto an affinity resin (AAVX) column for purification of the rAAV.

Capsids were eluted with a developed low pH buffer and subsequently neutralized followed by loading onto cesium chloride gradient sample tubes. Tubes were centrifuged at approximately 220,000 × *g* for a minimum of 16 h under temperature control using a Beckam Coulter Optima XE-90 Ultracentrifuge. Full vector and empty capsid fractions were purified using a 21-gauge needle, formulated in a PBS containing buffer, additionally processed to achieve the target VG/mL (or capsid particles [CP]/mL for the empty capsid fraction), and aliquoted until subsequent testing. Test articles were stored at −80°C until used.

### Size exclusion chromatography with UV and multiangle light scattering

An Agilent 1260 Infinity II LC system (Agilent, Santa Clara, CA) with binary pump, multisampler maintained at 4°C, multicolumn thermostat maintained at 25°C, and diode array detector was coupled to a DAWN HELEOS multiangle light scattering and Optilab refractometer for detection (both from Wyatt Technology Corporation, Santa Barbara, CA). An isocratic mobile phase of 1 × PBS (from 10 × PBS) containing 0.01% (vol/vol) pluronic F-68 was used after passing through a 0.2 μm filter. Liquid chromatography-mass spectrometer grade water was used for dilution of stock solutions. Two separate SEC analytical columns were used: Wyatt 5 μm 500Å 4.6 mm ID × 300 mm (Wyatt Technology Corporation catalog WTC-050N5) and a SRT 5 μm 1,000Å 4.6 mm ID × 300 mm (Sepax Technologies, Newark, DE; catalog 215950-4630).

Columns were equilibrated for ∼20 h before injection and flow rates (0.3 mL/min) and injection volumes (45 μL) were kept constant for direct comparisons. Absorbance at 260 and 280 nm were performed during testing and capsid and vector extinction coefficients for STRX-330 were determined using Optilab and UV detection. The extinction coefficients at 260 and 280 nm for the STRV84 were 1.40 and 2.01. In addition, the extinction coefficients at 260 and 280 nm for STRX-330 were 25.09 and 14.56. Peak detections, molar mass determinations and peak statistics were performed in ASTRA 8.1.1.

For assessment of analytical performance of SEC-MALS, linearity was performed by diluting the process confirmation test article across nine levels with triplicate volume transfers and singlicate injections. To achieve accuracy, five volume transfers at seven different levels were performed. Mobile phase was used as the diluent. For routine testing of rAAV, 10 μL of sample was diluted into 40 μL of mobile phase. Sample volumes were added into 2 mL polypropylene vials with 0.2 mL glass insert with vial screw caps (Agilent). Triplicate injections of BSA were used as a system check during each run.

### ddPCR and stunner testing

Design of experiment (DOE) approaches were used to develop and optimize a titer assay for the genome quantification of STRX-330. Multiple sets of primers and TaqMan probes were ordered from Invitrogen, and conditions were screened for desired response factors. The developed assay included a single set of primers with probe, a minimum of three serial dilution preparations of samples and five levels prepared in the presence of pluronic, treatment with Turbo DNase (Ambion), heat and chelator inactivation of the DNase, and dilution in water. DNase treated, diluted samples were mixed with ddPCR supermix for probes and gene-specific primers/probe. Droplets were generated using an Auto-droplet generator. Plates were sealed with a PX1 plate sealer and PCR cycling was performed with C1000 Touch Thermal cyclers. Droplets were read with a QX200 Droplet Reader using QX Manager software. Data were analyzed and VG/mL values determined using an internally developed Microsoft Excel spreadsheet.

Samples were analyzed by Stunner using the Gene Therapy application with the AAV Quant workflow. The Stunner instrument collects UV/Vis, dynamic light scattering (DLS), and static light scattering (SLS) data from a single sample well. The theoretical molar masses for both the capsid and the target packaged DNA were included in the software parameters. A minimum of triplicate volume transfers were performed to directly measure CQAs using the Stunner low-volume 96-well plates.

### Capsid ELISA

DOE approaches were used to develop and optimize a sandwich ELISA method for capsid quantification of STRX-330. Streptavidin was used to coat Maxisorp nunc plates. Coated plates were blocked with Blocker Casein in PBS. CaptureSelect Biotin AAV9 and CaptureSelect HRP AAV9 were used for capture and detection steps, respectively. rAAV samples were diluted into the analytical range for the assay using 1 × PBS Tween (diluted from 10 × ). TMB substrate was used for HRP detection and the reaction was stopped by addition of diluted sulfuric acid. Optical density at 450 nm was performed using an iD5 multimode plate reader and blank corrected optical density (OD) values were used to determine concentrations (Molecular Devices, San Jose, CA).

Standard curve material consisted of empty capsids of the same serotype as STRX-330, produced and purified within StrideBio as above, and were quantified by SEC-MALS. Sample values were regressed against the standard curve using a 5-parameter logistic curve fit with data analysis performed within Softmax Pro 7.1.0. ELISA plates were washed using 1 × PBS Tween with a plate washer (Agilent) and incubation steps of the method were temperature controlled using Benchmark Scientific Incu-Mixer MP4 (Sayreville, NJ).

### Sodium dodecyl sulfate-capillary electrophoresis

Protein purity was assessed using a SCIEX PA800 plus capillary unit with a preassembled bare-fused silica capillary cartridge (SCIEX, Framingham, MA). Reagents (basic/acid washes and sodium dodecyl sulfate [SDS] MW gel buffer) from an SDS-MW analysis kit were used (SCIEX). When needed, LC-MS grade water was used. CE was added to inlet and outlet trays in standard CE vials (SCIEX). Samples were added to sample trays in nano CE vials (SCIEX).

Samples were denatured in 0.2% SDS (from ultrapure 10% SDS) at 75°C for 10 min. Following a conditioning run, samples were electrokinetically injected at 5.0 kV for 99.9 s and separated at 10.0 kV for 50 min. Similar concentrations of each test article (∼2 × 10^12^ CP/mL) were analyzed and the corrected areas as well as VP ratios were determined. Six independent volume transfers were performed and each replicate was measured with a single injection. Peak detections and performance were performed in 32Karat software with peak resolution and asymmetry calculated according to USP guidance.

### TCID_50_ assay

Development and optimization of the classical TCID_50_ assay^34,35^ was performed before testing. In brief, HeLaRC32 cells were cultivated in DMEM with 10% FBS for ∼3–5 days in T75 culture flasks before plating. Cells were trypsinized, counted with a Cedex HiRes Analyzer (Roche, Basel, Switzerland) and 50 μL at 8.0 × 10^5^ cells/mL plated on Falcon polystyrene 96-well tissue culture plates (Corning). Plates were incubated overnight at 37°C/CO_2_ incubator before use. An infection medium preparation was performed by combining DMEM, 0.01% pluronic, 25 mM HEPES, and adenovirus to 3.2 × 10^8^ particles/mL. AAV samples were diluted in infection medium to the target analytical multiplicity of infection range. Two-fold assay plate dilutions were performed with four independent volume transfers for each cell assay plate. A single column of no AAV addition was used to monitor well-to-well contamination.

Control AAV material was prepared in the same manner and included on each assay plate. Cell culture medium was aspirated from the plated HeLaRC32 cells and was washed with DPBS. Fifty microliters of the serially diluted samples in infection medium were added to the plates and the plates were incubated for 2 h at 37°C/CO_2_ conditions. Fifty microliters of complete medium was added and the plates were incubated for ∼3 days. DNA was extracted using a solution composed of proteinase K buffer (1 mM Trizma with 1 mM EDTA and 0.1% SDS), Tween 20, deoxycholate, and 1 × PBS. Eighty-five microliters of the extraction solution was added to all wells of the assay plate followed by incubations at 37°C for 1 h, 55°C for 2 h, and 30 min at 95°C. Treated plates were sealed and stored at 2–8°C for up to 7 days before analysis.

DNA signal was quantified using ddPCR. In brief, extracts from TCID_50_ assay plates were analyzed by diluting 100-fold in water with pluronic acid. Droplet generation, PCR cycling, and droplet reading were performed as above without a DNase treatment step. Data were analyzed using an internally developed Microsoft Excel spreadsheet. The copies/μL values were measured and the background signal (from the no AAV wells) was averaged and subtracted from sample/control wells. The total number of copies per well was determined and the input VGs were subtracted. Wells were scored as positive or negative and the TCID_50_/mL and VG particle to infection ratios were determined using the Spearman-Kärber's method from publicly available spreadsheets that were modified for use.^35^

### Sedimentation velocity analytical ultracentrifugation

Purified vector from the 500 L production was considered as the full vector material. In addition, the empty capsid fraction from this production was also separately purified. A six-point curve was generated using the full vector (sample 1) followed by spiking the full vector with volumes of empty capsid material (samples 2 through 5). In addition, the empty capsid material was included in the data collection (sample 6). Samples 1–6 were analyzed by SV-AUC at BioAnalysis, LLC (Philadelphia, PA). In brief, formulation buffer was used for sample dilution to a final concentration range of 0.5–0.9 OD at 230 nm. Samples with lower than 0.5 OD were analyzed without dilution. Four hundred twenty microliters was loaded into the reference and 410 μL into the sample sectors (two sector charcoal, Epon centerpiece; quartz/sapphire windows).

The samples were allowed to achieve temperature equilibrium 1.5 h after the vacuum stabilized at 0 μm. The experiments were collected using a Beckman Coulter XLI and sedimentation was followed at 230 nm. Rotor speed was 12,000 rpm and the scanning frequency was set to 150–200. SEDFIT (16.36) was used to generate the c(s) and ls-g*(s) distributions from the SV-AUC data. Figures were generated using GUSSI (1.4.2). SEDNTERP (v.3.0.3; March 14, 2021) was used to determine the density and viscosity of the buffer. Sedimentation coefficients were uncorrected (s,S) for buffer, water, and 20°C. Integration of the c(s) distributions was performed using both GUSSI and OriginLab (2015) or directly in Excel.

### Cryogenic electron microscopy

The dilution series of full and empty capsids from above were analyzed by cryo-EM at the University of Florida ICBR Electron microscopy core. In brief, for each sample, 3.5 μL was applied to a glow-discharged Quantifoil copper grid with 2 nm continuous carbon support over holes (Quantifoil R 2/4 400 mesh), blotted, and vitrified using a Vitrobot Mark 4 (FEI) at 95% humidity and 4°C. Images were collected using an FEI Tecnai G2 F20-TWIN microscope (FEI) operated under low-dose conditions (200 kV, ∼20e−/Å2) on a GatanUltraScan 4000 CCD camera (Gatan). The number of empty and full capsids in these images were counted manually.

### pH stress studies

The study of STRX-330 in response to pH stress conditions was performed using concentrated HCl or NaOH. In brief, vials of STRX-330 were removed from −80°C and thawed overnight at 4°C. To initiate the stress conditions, HCl or NaOH was added to achieve a final concentration of 0.01 N. The volume of acid or base was ∼1% of the sample volume to avoid dilution of formulation buffer. Samples were kept at room temperature for 3 h and then neutralized with the opposite treatment. For instance, acid-treated samples were neutralized with equimolar base and *vice versa*. As a control, equimolar base and acid were mixed to neutralize and then added to STRX-330. The final pH of the stressed conditions were ∼4.0 for acid treated, ∼11 for base treated, and ∼7.5 for neutralized treated.

### Data analysis and documentation

Data sets were analyzed in Microsoft Excel and GraphPad Prism (9.0). Graphs were generated with GraphPad Prism (linear regression) and coefficient of variance (%CV) was determined using Excel. Experiments were documented in near-time within the electronic laboratory notebook of StrideBio.

## RESULTS

### Method development for SEC-MALS

**Figure 1.**
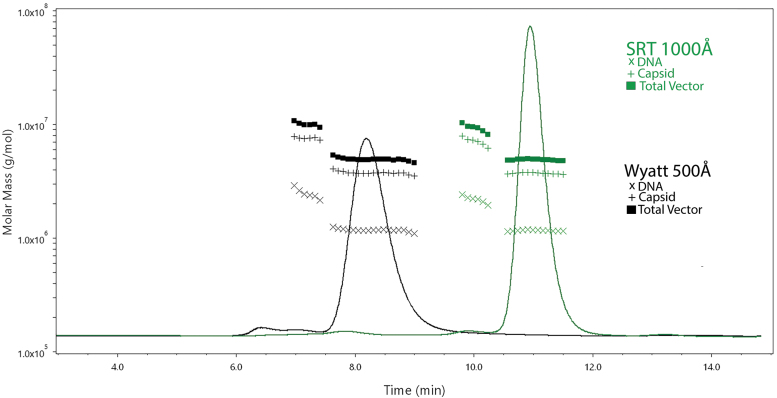
Representative SEC-MALS UV chromatograms with molar mass information obtained with two separate columns. Samples were diluted fivefold in mobile phase and 45 μL was injected. Flow rate was 0.3 mL/min and the multisampler was maintained at 4°C. Ten separate volume transfers were performed from a single vial of process confirmation material. Data shown are from a representative chromatogram and the total data from 10 independent injections are shown in Supplementary Table S1. The *black trace* was obtained with the Wyatt 500Å column and the *green trace* was obtained with the SRT-1,000Å column. Baseline-to-baseline integration of the monomer peak was performed in ASTRA software to obtain protein, DNA, and peak statistics. The test article was the process confirmation material. The molar masses for the DNA, capsid, and total vector symbols are indicated in figure. SEC-MALS, size exclusion chromatography with UV and multiangle light scattering.

Development of the method for testing STRX-330 was performed using an isocratic mobile phase with two separate SEC analytical columns, a Wyatt brand SEC column (5 μm, 500Å) and a Sepax Technologies SRT SEC column (5 μm, 1,000Å). A representative chromatogram from these injections is shown in Fig. 1.

Monomeric AAV elution times were ∼8.5 and ∼11 min for the Wyatt and SRT columns, respectively. The dimer peak eluted at ∼7 min for the Wyatt column and ∼10 min for the SRT column. Interestingly, there was third peak that eluted at ∼6.5 min for the Wyatt column and ∼7.5 min for the SRT column. While the monomeric and dimer peaks both exhibited VG/CP ratios consistent with AAV, this third peak exhibited pronounced absorbance at 260 nm with minimal absorbance at 280 nm. Importantly, the monomeric peak statistics demonstrated acceptable analytical performance for asymmetry, tailing factor, and resolution between the dimer peak when either column was used (Supplementary Table S1).

Both columns demonstrated strong agreement in vector titer (capsid and genome), vector molar mass (within 1% of theoretical), and DNA molar mass without any additional optimization required (Supplementary Table S1). These data sets were compared to internally developed ddPCR and capsid ELISA methods and are shown in Supplementary Table S2. The DNase-resistant mean ddPCR value was within 20% while the mean capsid ELISA value was within 5% of the SEC-MALS VG/mL and CP/mL measurements, respectively. Comparable to SEC-MALS precision, both ddPCR and ELISA values produced intra-assay measurements <3%. The Wyatt 500Å column was used for subsequent experiments.

**Figure 2.**
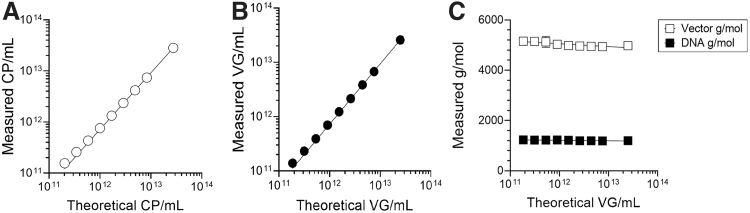
Linearity of CP/mL, VG/mL, and molar mass SEC-MALS data obtained with the Wyatt 500Å column. Samples were diluted twofold followed by serial 1.7-fold dilutions to cover nine levels. Five volume transfers were performed at each level with singlicate injections (injection volume and flow rate were as in Fig. 1). Baseline-to-baseline integration of peaks were performed in ASTRA software to obtain protein, DNA, and peak statistics. **(A)** Mean CP/mL concentrations of injected material at each dilution level from five preparations at each level are shown. Error bars show the standard deviation, but are below visibility on the graph. **(B)** Mean VG/mL concentrations of injected material at each dilution level from five preparations at each level are shown. Error bars show the standard deviation, but are below visibility on the graph. **(C)** Mean molar mass data for vector and DNA content of injected material at each dilution level from five preparations at each level are shown. Error bars show the standard deviation. Test article was the process confirmation material. CP, capsid particles; VG, vector genomes.

To determine whether the SEC-MALS approach could achieve ICH and USP recommendations for analytical methods, linearity and accuracy by recovery tests were performed. To determine linearity of a method, a minimum of five concentrations distributed across the range of detection is recommended.^36^ The mean CP/mL and VG/mL values shown in Supplementary Table S1 obtained with the Wyatt column were used to assess linearity and accuracy by recovery. A plot of the theoretical CP/mL and VG/mL against the measured values produced strong linearity (*R*^2^ > 0.999) over two orders of magnitude (Fig. 2A, B). Moreover, the mean molar mass data for both vector and DNA were consistent across these sample ranges (Fig. 2C). Accuracy was assessed by using an appropriate number of determinations and levels that cover the expected reportable range.^36^

**Figure 3.**
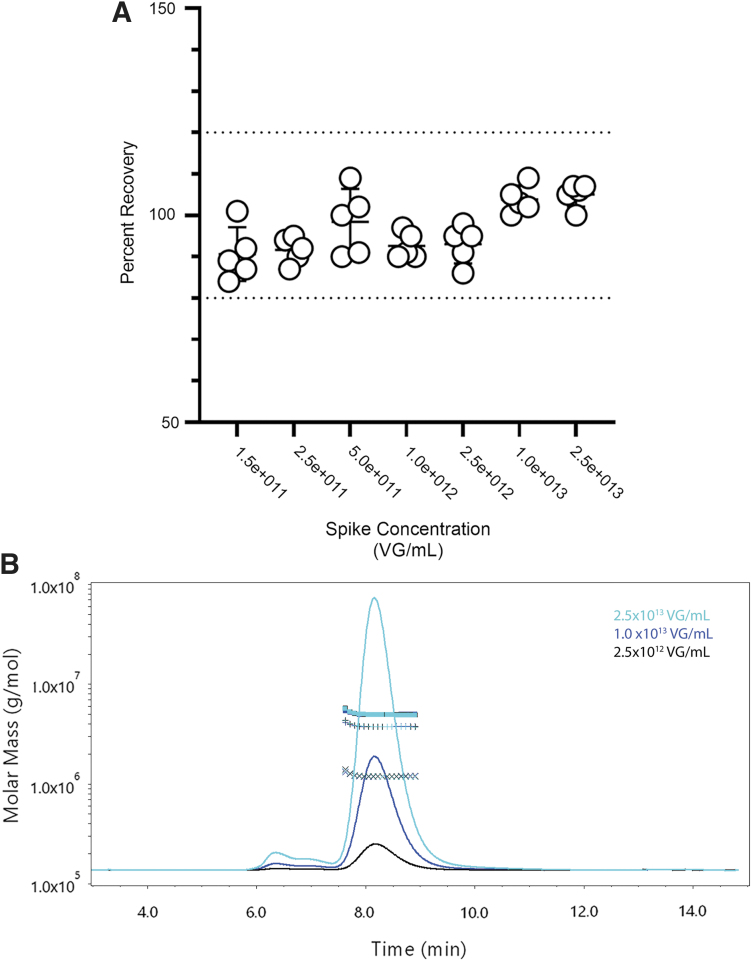
VG/mL accuracy by recovery using the Wyatt 500Å column. **(A)** Five volume transfers at each level were performed with the process confirmation material. The mean VG/mL value from Supplementary Table S1 was used to establish the accepted VG/mL value of the sample. The recovery of each injection at each level is shown with the mean ± standard deviation. A *dashed line* shows ±20% of 100% recovery. The complete tabulated data with molar mass information is shown in Table 1. **(B)** Representative UV chromatogram from SEC-MALS data in **(A)**. Data from the three highest spike levels are shown. The *cyan*, *blue*, and *black traces* correspond to the 2.5 × 10,^13^ 1.0 × 10,^13^ and 2.5 × 10^12^ VG/mL spike concentrations, respectively. The y-axis is the relative scale from the UV 260 nm trace. The aggregate peaks are detectable near 6.3 to ∼7 min elution times. Injection volumes and flow rates were as in Fig. 1. The molar masses for the DNA, capsid, and total vector symbols are as in Fig. 1.

Each replicate recovered within 20% of the spike value across all levels (Fig. 3A; Table 1). There was an increase in the variability for molar masses and concentrations below 1.0 × 10^12^ VG/mL (Table 1). Since one of the goals of SEC-MALS is to detect and characterize aggregate species, it was also evident that the off-target peaks were at low concentrations (<4% of total mass) and required higher sample concentrations (>2.5 × 10^12^ VG/mL; Fig. 3B). To achieve accurate and precise measurements for all CQAs of the monomer peak with SEC-MALS, these results would indicate that our analytical range for the assay is between >2.5 × 10^12^ and 2.5 × 10^13^ VG/mL. This method was used to compare against other methods of quantifying full-to-empty ratios with STRX-330 purified from a 500 L production run.

**Table 1.** Accuracy by recovery data by size exclusion chromatography with UV and multiangle light scattering

Spike Concentration (VG/mL)	Mean Measured VG/mL	%CV	Mean Percent Recovery	Mean Vector Molar Mass (g/mol)	%CV	Mean DNA Molar Mass (g/mol)	%CV	Mean VG/CP Ratio
1.50E+11	1.36E+11	7%	91%	5.24E+06	5%	1.28E+06	5%	0.950
2.50E+11	2.29E+11	3%	92%	5.13E+06	1%	1.23E+06	2%	0.916
5.00E+11	4.91E+11	8%	98%	5.16E+06	4%	1.25E+06	4%	0.926
1.00E+12	9.28E+11	3%	93%	5.02E+06	1%	1.22E+06	1%	0.927
2.50E+12	2.32E+12	5%	93%	5.03E+06	1%	1.22E+06	1%	0.922
1.00E+13	1.04E+13	4%	104%	5.00E+06	0%	1.21E+06	0%	0.918
2.50E+13	2.63E+13	3%	105%	4.98E+06	0%	1.20E+06	0%	0.916

%CV, coefficient of variance; CP, capsid particles; VG, vector genomes.

### Comparison of various methods in the quantification of full to empty rAAV particles

Using purified material from a 500 L production, multiple methods were used to quantify the full-to-empty particles of a preclinical stage gene therapy program. A dilution series was generated that ranged from undiluted full vector (sample 1), full vector spiked with varying volumes of empty capsids (samples 2–5), and undiluted empty capsids (sample 6). This approach generated a 6-point curve of varying full-to-empty particles. The full vector was used in a non-GLP animal study as part of program development (STRX-330). Sufficient volumes of samples 1–6 were obtained to allow for a single dilution curve to be analyzed by all methods. To avoid complicating the interpretation of data from mixing samples of varying purity, each test article was assessed by denaturing SDS-capillary gel electrophoresis (SDS-CE).

A representative result is shown in Supplementary Fig. 1 and the compiled data are shown in Supplementary Table S3. As observed with other AAV serotypes, both the full vector and the empty capsid preparations displayed the truncated VP3, also referred to as VP3 variant.^37,38^ Corrected areas for VPs achieved acceptable precision from repeatability testing (%CV ≤10%) with similar VP1:VP3 and VP2:VP3 ratios for both test articles. Collectively, the two test articles displayed similar purity profiles and were considered similar enough to generate a dilution series for full-to-empty quantifications.

The dilution curve of full and empty capsids was prepared to ∼1.5 × 10^13^ CP/mL and the levels of full, partial, and empty capsids were measured via SV-AUC, cryo-EM, SEC-MALS, ddPCR/ELISA, and Stunner. The complete data set comparing methods is shown in Supplementary Table S4. For SV-AUC, the sedimenting boundaries and resulting c(s) distribution for samples 1 through 6 are shown in Supplementary Fig. S2. The full vector comprised 89.9% of the total signal at sedimentation coefficient of 101.2 S. As expected, the empty capsid comprised 90.1% of the total signal at 62.9 S. The dilution curve demonstrated a linear response with the measured full-to-empty ratio via SV-AUC (Table 2). The level of partial genomes was ≤5% for all samples tested demonstrating a low level of this population obtained from the 500 L production.

**Table 2.** Sedimentation velocity analytical ultracentrifugation data from full-to-empty dilution curve

SV-AUC Data					
	LOC (34.1–48.4 S)	HOC (134.7–140.7 S)	Empty Capsids (62.9–66.6 S)	Partial Capsids (80.9–91.8 S)	Full Capsids (101.2–101.7 S)
Sample 1 (full)	0.7%	5.7%	0.6%	3.1%	89.9%
Sample 2	0.6%	1.1%	17.6%	3.0%	77.7%
Sample 3	0.7%	1.2%	34.1%	2.9%	61.1%
Sample 4	0.5%	5.2%	47.6%	3.6%	43.1%
Sample 5	0.6%	1.4%	62.7%	4.3%	31.1%
Sample 6 (empty)	0.7%	1.7%	90.1%	3.7%	3.8%

HOC, higher order capsids; LOC, lower order capsids; SV-AUC, sedimentation velocity analytical ultracentrifugation.

Although SV-AUC is often used for the measurement of full-to-empty AAV, this approach is capable of quantifying additional traits such as lower order capsids (LOC) and higher order capsids (HOC). LOC and HOC may contain low molecular weight contaminants or aggregated capsids, respectively. For these samples, the LOC were ≤1% and the HOC were ≤6% (Table 2).

**Figure 4.**
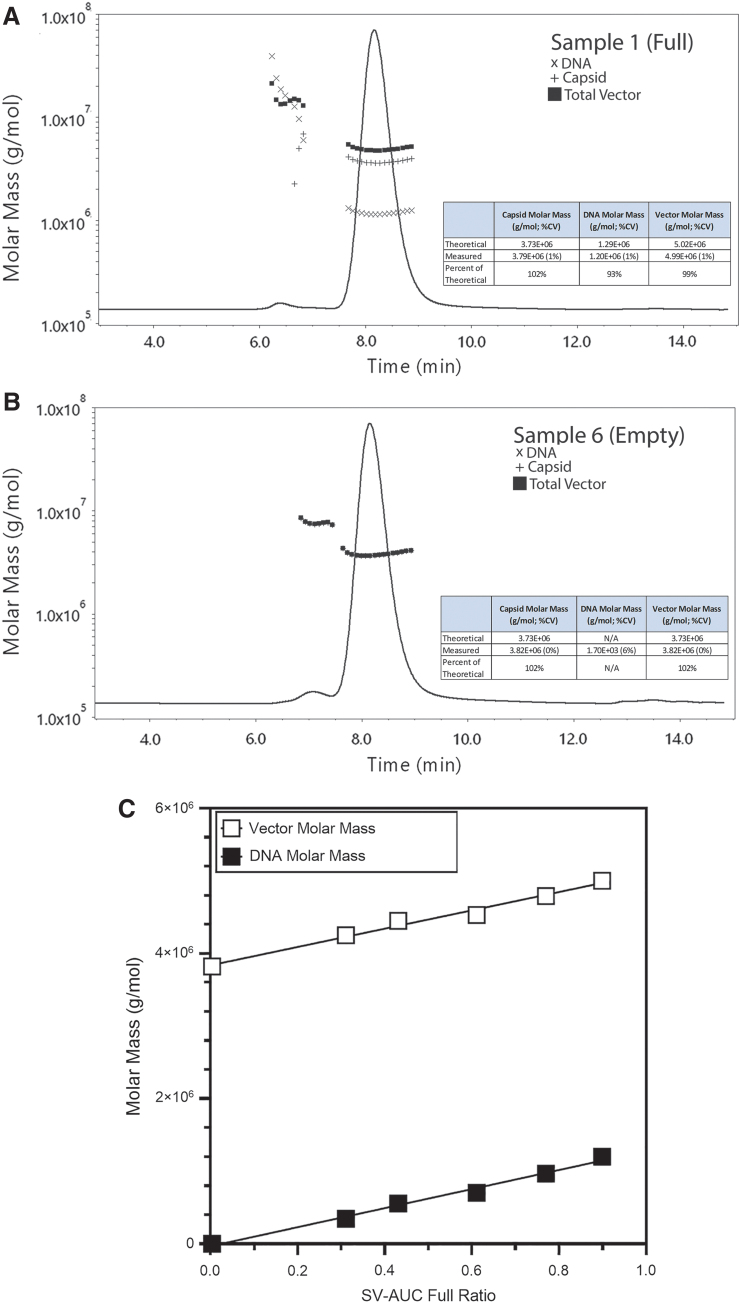
SEC-MALS data from the dilution series of full and empty particles to generate a curve of varying full-to-empty particles. **(A)** Full vector material from a 500 L production run was diluted fivefold using five separate volume transfers. A representative UV trace with molar mass data is shown. The *inset* table shows the mean values across the five injections and percent of measured value compared to the theoretical value for molar masses. The molar mass data from a peak (∼6.5 min elution time) with 260 nm value well above the 280 nm measurement are also shown. The *parenthesis* lists the %CV value across the five injections. **(B)** Representative UV trace from the empty capsid material is shown. Samples were prepared as in **(A)** and the *inset* table shows the mean molar mass values from each of the target molecule (protein and DNA). The *parenthesis* lists the %CV value across the five injections. A presumptive dimer peak is shown that eluted at ∼7 min is shown. **(C)** A linearity plot of the total molar mass data across the varying full-to-empty particle dilution curve relative to the SV-AUC full ratio is shown. The mean molar mass values are shown with error bars displaying the standard deviation, but are below visibility on the graph. The *R*^2^ value shows the linear regression for both target molecules (protein and DNA). Injection volumes and flow rates were as in Fig. 1. The complete tabulated data are shown in Table 3. %CV, coefficient of variance; SV-AUC, sedimentation velocity analytical ultracentrifugation.

**Figure 5.**
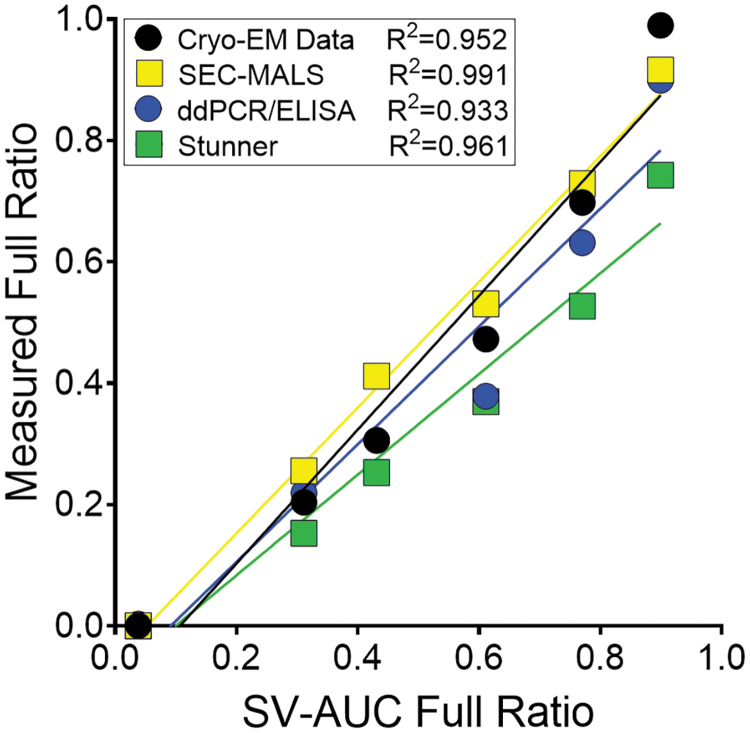
Linear regression data for all tested methods compared to the SV-AUC full ratio values. The value of full material quantified across the full-to-empty dilution curve by each method shown against the SV-AUC value is shown. In the case of cryo-EM, the single value for each point are shown, but for SEC-MALS, ddPCR/ELISA, and Stunner, the mean full value is shown. The *R*^2^ value shows the linear regression using the full ratio. For the data points near 0.4 SV-AUC Full Ratio, the ddPCR/ELISA and cryo-EM data overlap. The tabulated data are shown in Supplementary Table S4. ELISA, enzyme-linked immunosorbent assay; cryo-EM, cryogenic electron microscopy; ddPCR, droplet digital PCR.

Cryo-EM data produced similar results for the full and empty preparations (Supplementary Fig. S3A–C; Supplementary Table S4); however, cryo-EM measured the full vector at 99% full versus 89.9% for SV-AUC. When compared to SV-AUC, only the first two samples on the dilution curve measured within 20% of the corresponding SV-AUC value (Supplementary Table S4). There was a trend with cryo-EM that suggested a bias toward the quantification of empty capsids over full capsids. Nevertheless, the cryo-EM results were linear with respect to the SV-AUC values (*R*^2^ = 0.952; Fig. 5). SEC-MALS data demonstrated full-to-empty values close to SV-AUC and the measured molar masses were within 5% of the theoretical values for the full and empty preparations, respectively (Table 3; Fig. 4A, B).

**Table 3.** Size exclusion chromatography with UV and multiangle light-scattering data from full-to-empty dilution curve

SEC-MALS Data					
	Mean Vector Molar Mass (g/mol)	%CV	Mean DNA Molar Mass (g/mol)	%CV	Mean VG/CP Ratio
Sample 1 (full)	4.99E+06	1%	1.20E+06	1%	0.916
Sample 2	4.79E+06	1%	9.66E+05	1%	0.729
Sample 3	4.53E+06	0%	7.03E+05	1%	0.531
Sample 4	4.45E+06	1%	5.56E+05	1%	0.412
Sample 5	4.25E+06	1%	3.45E+05	1%	0.256
Sample 6 (empty)	3.82E+06	0%	1.70E+03	6%	0.001

SEC-MALS, size exclusion chromatography with UV and multiangle light scattering.

With the exception of the empty sample, all SEC-MALS VG/CP ratios were within 20% of the SV-AUC values. In addition, the SEC-MALS data exhibited strong linearity with SV-AUC data (Fig. 5; *R*^2^ = 0.991). When plotted against the SV-AUC full ratio, the vector and DNA molar masses were linear (Fig. 4C).

Of the methods tested here, ddPCR is the only approach that utilizes a DNase treatment before quantification. When coupled with a capsid ELISA, the quantification of both genome and capsid concentrations is obtained. A developed and optimized titer assay specific to the genome content of STRX-330 was utilized. In addition, an optimized sandwich ELISA was performed for capsid quantification. Both workflows were performed in parallel using the same intermediate sample dilutions. Except for one sample in the dilution curve, the interassay precision was ≤15% for both capsid and genome concentrations (Table 4).

**Table 4.** Quantification of capsid particles/mL and vector genomes/mL from size exclusion chromatography with UV and multiangle light scattering, droplet digital PCR/enzyme-linked immunosorbent assay, and stunner

SEC-MALS					
	CP/mL	%CV	VG/mL	%CV	VG/CP Ratio
Sample 1 (full)	1.77E+13	1%	1.62E+13	1%	0.916
Sample 2	1.31E+13	3%	9.54E+12	3%	0.729
Sample 3	1.49E+13	1%	7.91E+12	1%	0.531
Sample 4	1.06E+13	1%	4.37E+12	1%	0.412
Sample 5	1.27E+13	1%	3.25E+12	2%	0.256
Sample 6 (empty)	9.25E+12	0%	1.02E+10	8%	0.001

ddPCR/ELISA (Operators 1 and 2)					
	Capsid ELISA		ddPCR VG/mL		Full Ratio
	CP/mL	%CV	VG/mL	%CV	
Sample 1 (full)	1.69E+13	2%	1.52E+13	13%	0.900
Sample 2	1.43E+13	9%	9.01E+12	3%	0.632
Sample 3	1.82E+13	26%	6.89E+12	15%	0.379
Sample 4	1.33E+13	2%	4.07E+12	6%	0.306
Sample 5	1.24E+13	4%	2.73E+12	6%	0.219
Sample 6 (empty)	1.36E+13	11%	4.72E+10	13%	0.003

Stunner					
	CP/mL	%CV	VG/mL	%CV	Full Ratio
Sample 1 (full)	2.02E+13	1%	1.50E+13	0%	0.744
Sample 2	1.91E+13	2%	1.01E+13	2%	0.528
Sample 3	1.82E+13	3%	6.73E+12	3%	0.369
Sample 4	1.82E+13	3%	4.63E+12	2%	0.254
Sample 5	1.89E+13	3%	2.90E+12	8%	0.154
Sample 6 (empty)	1.73E+13	12%	ND		

ddPCR, droplet digital PCR; ELISA, enzyme-linked immunosorbent assay; ND, not detected.

Compared to the capsid and genome concentrations obtained by SEC-MALS, mean CP/mL values were within 30% across the sample curve; however, VG/mL values were within 20% and ddPCR was capable of accurately quantifying the concentration of the target genome within the empty preparation (Table 4). The full-to-empty ratio for the full preparation was 90% and strikingly close to the SV-AUC value of 89.9%. Apart from one sample, the full-to-empty ratio was within 30% of the SV-AUC. ddPCR/ELISA results were linear compared to the SV-AUC value (*R*^2^ = 0.933; Fig. 5). Compared to the data from SEC-MALS, all samples tested by ddPCR/ELISA were within 30% of the full-to-empty ratio (Supplementary Table S4).

Bulk absorbance methods have been used to quantify AAV capsid and genomes.^17^ The Stunner instrument from Unchained Labs offers the combination of UV/Vis measurements coupled with DLS and SLS. Similar to SV-AUC, cryo-EM, and SEC-MALS, this approach is capable of measuring multiple CQAs. A major benefit of the Stunner instrument is a small sample requirement (2 μL) with minimal sample preparation, which allows for an orthogonal method to titration assays with minimal impact on the sample consumption. With the exception of the full sample, all full-to-empty values obtained with the Stunner were more than 30% lower than the corresponding SV-AUC value (Supplementary Table S4). Because the full-to-empty value for SEC-MALS, ddPCR/ELISA, and Stunner are based on the measurement of both capsid and genome concentrations, measurements from these three approaches were compared.

The VG/mL values across all three methods were very similar with each level from SEC-MALS and Stunner within 20% of the DNase-resistant ddPCR value (Table 4). This indicates that each method is accurate for the quantification of AAV genomes with purified samples. The concentration of capsids with these three methods exhibited more variance. The CP/mL values with each level from SEC-MALS and Stunner were within 50% of the ELISA value (Table 4). SEC-MALS measurements were closer to the ELISA values and Stunner CP/mL measurements tended to overestimate the number of capsids present. Nevertheless, full-to-empty data from Stunner were linear when compared to SV-AUC (*R*^2^ = 0.961; Fig. 5).

### Stability-indicating performance of SEC-MALS and ddPCR/ELISA

Given the alignment of SEC-MALS with SV-AUC full-to-empty data and the ability to quantify CP/mL and VG/mL, we conducted a series of experiments to assess the method's stability-indicating performance. To test this, the STRX-330 material used for the non-GLP animal study was subjected to pH stress at room temperature for 3 h. Aliquots of the test article were treated with HCl or NaOH to a final concentration of 0.01 N. As a control, the concentrated HCl and NaOH were premixed to neutralize and then added to a separate aliquot. After 3 h of incubation, the opposing treatment was added to neutralize the pH and stop the stress. For instance, the 0.01 N HCl treated sample received equimolar NaOH to neutralize. Then, the samples were tested by SEC-MALS, ddPCR/ELISA, and infectivity was assessed by TCID_50_.

**Figure 6.**
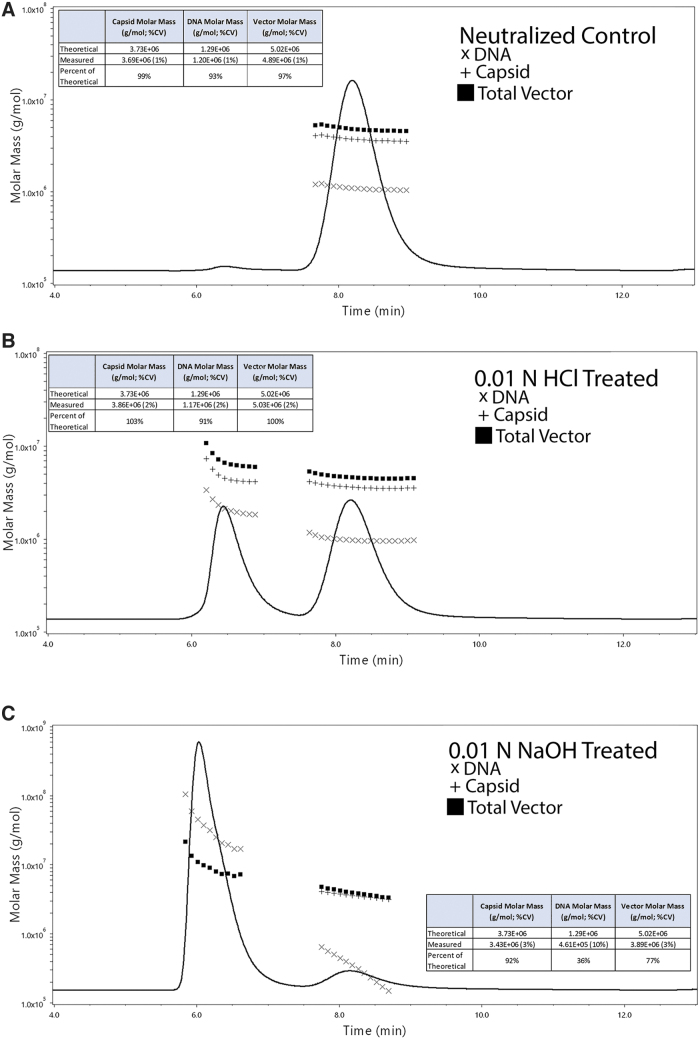
Impact of pH stress on STRX-330 as measured by SEC-MALS. Aliquots of STRX-330 purified from a 500 L production run were treated with either (1) an equimolar solution of HCl and NaOH, (2) 0.01 N HCl, or (3) 0.01 N NaOH for 3 h at room temperature. **(A)** Representative UV chromatogram displaying the molar masses for the neutralized treated sample that was diluted fivefold using five separate volume transfers after the 3-h incubation. The *inset* table shows the mean values across the five injections and percent of measured value compared to the theoretical value for molar masses. The *parenthesis* lists the %CV value across the five injections. **(B)** Representative UV chromatogram displaying the molar masses for the 0.01 N HCl-treated sample. The *inset* table shows the mean values across the five injections and percent of measured value compared to the theoretical value for monomer molar masses. The *parenthesis* lists the %CV value across the five injections. The molar mass data from the aggregated peak around 6.5 min is also shown. **(C)** Representative UV chromatogram displaying the molar masses for the 0.01 N NaOH-treated sample. The *inset* table shows the mean values across the five injections and percent of measured value compared to the theoretical value for monomer molar masses. The *parenthesis* lists the %CV value across the five injections. The molar mass data from the aggregated peak around 6.0 min are also shown. Injection volumes and flow rates were as in Fig. 1. The complete tabulated VG/mL and CP/mL data are shown in Table 5.

TCID_50_ and ddPCR/ELISA were used since these are known to indicate capsid function and can be compared with results from SEC-MALS. As expected, the control treated material exhibited SEC-MALS profile similar to untreated and earlier tested samples (Fig. 6A). However, treatment with either HCl or NaOH resulted in very different chromatograms. Specifically, the HCl treatment produced two major peaks; one corresponding to the monomer peak and the other corresponding to an aggregated peak (Fig. 6B). Treatment with NaOH dramatically altered the SEC elution profile of AAV with a pronounced reduction in the monomer peak. Instead, a prominent peak eluting slightly before the typical aggregated peak followed by a minor monomer peak was observed (Fig. 6C).

In response to alkaline stress, there was a clear shift in the population of nucleic acid content (determined from the molar mass data) within the monomer peak. Since the protein molar mass did not change, this would suggest that an alkaline pH condition has a profound impact on the encapsidated DNA content. The acidic pH stress condition impacted the capsid more so than DNA by promoting aggregate formation. Surprisingly, the ddPCR/ELISA data for the HCl-treated sample exhibited only a modest reduction in the CP/mL and DNase-resistant VG/mL values. However, the NaOH-treated material reduced the CP/mL (∼twofold) and the VG/mL values (>20-fold; Table 5). Following treatment with 0.01N HCl, TCID_50_ data demonstrated only a modest change in the infectivity. Following treatment with 0.01 N NaOH, infectivity measured by TCID_50_ was markedly reduced to undetectable levels (Table 5).

**Table 5.** Size exclusion chromatography with UV and multiangle light scattering results from pH stress compared with other stability-indicating assays

SEC-MALS					
Treatment	CP/mL	%CV	VG/mL	%CV	VG/CP Ratio
Neutralized Control	4.60E+13	3%	4.29E+13	3%	0.933
0.01 N HCl	2.72E+13	1%	2.37E+13	1%	0.873
0.01 N NaOH	1.24E+13	10%	4.14E+12	5%	0.336

ddPCR/ELISA					
Treatment	Mean CP/mL	%CV	Mean VG/mL	%CV	VG/CP Ratio
Neutralized Control	4.54E+13	7%	4.58E+13	3%	1.008
0.01 N HCl	3.35E+13	13%	2.61E+13	2%	0.779
0.01 N NaOH	2.75E+13	6%	1.73E+12	11%	0.063

TCID_50_					
Treatment	TCID_50_/mL		Mean TCID_50_/mL	%CV	Mean P:I Ratio
	Operator 1	Operator 2			
Neutralized Control	4.93E+08	1.16E+09	8.28E+08	57%	7.13E+04
0.01 N HCl	2.57E+08	4.33E+08	3.45E+08	36%	1.24E+05
0.01 N NaOH	<8.00E+06	<8.00E+06	<8.00E+06	N/A	>5.00E+06

P:I, particle to infection; TCID_50_, 50% tissue culture infectivity.

## CONCLUSION

A successful gene therapy program requires robust and adaptable analytical capabilities. Primary areas of analytical focus for preclinical and early-stage programs are establishing the dose-defining assay(s), transduction assays to monitor expression of the DNA payload, and solidifying the safety and stability-indicating assays. Shown here is that SEC-MALS is a valuable analytical tool that assists in meeting these areas of need. A product-specific SEC-MALS method required little development and optimization and could achieve the recommended ICH/USP guidance for validation of chromatography methods. Comparison of full-to-empty methods demonstrated strong linearity and accuracy with values obtained by SV-AUC. The latter is considered the “gold standard” for quantification of full particles.

However, we note that agreement between SEC-MALS and SV-AUC may rely on the level of partial genome concentrations within the purified vector. AAV containing partial genomes may influence the SEC-MALS full-to-empty ratio leading to inaccurate measurements. In addition to SEC-MALS and other approaches, we believe that methods capable of quantifying partial genomes, such as SV-AUC,^18^ be utilized early in program development in accordance with an enhanced approach to analytical procedure development.

The VG/mL values obtained with SEC-MALS were within 20% of the DNase-resistant ddPCR values, which is considered the primary dose-defining assay for AAV. When used in combination with other stability-indicating assays, SEC-MALS was capable of providing insight into AAV degradation in response to pH stress. In addition to pH stress, 10 × freeze-thaws and temperature stresses were also performed. These data sets demonstrated that SEC-MALS was capable of detecting modest to more extreme changes in the AAV monomer peak (data not shown). This supports the utility of SEC-MALS early in program development since minimal sample handling and consumption are needed to generate analytical data that are supported with orthogonal methods.

Moreover, these results support that methods developed with SEC-MALS can meet the analytical testing guidelines and are capable of being transferred into a quality control laboratory. Perhaps more interesting is the ability of SEC-MALS to provide fundamental understanding of the response of AAV to environmental stress conditions. This was demonstrated with the pH stress studies that determine acidic and alkaline pH negatively impact capsid and genome content, respectively. This information may yield additional knowledge of how AAV responds to conditions experienced during its life cycle and the manufacturing process.

## Supplementary Material

Supplemental data

Supplemental data

Supplemental data

Supplemental data

Supplemental data

Supplemental data

Supplemental data
