# Supplementary material for: Application of Size Exclusion Chromatography with Multiangle Light Scattering in the Analytical Development of a Preclinical Stage Gene Therapy Program
Source: Hum Gene Ther. 2023 Apr 17;34(7-8):325–38. doi: 10.1089/hum.2022.218 (PMC10125404; doi:10.1089/hum.2022.218)
Supplement: Supplemental data [file Supp_TableS1.docx]

**Supplemental Data Table 1** – Peak Statistics From SEC-MALS Characterization Data with Two SEC Columns

| **Wyatt 500Å** | | | | | | | |
| --- | --- | --- | --- | --- | --- | --- | --- |
| **Replicate** | **CP/mL** | **VG/mL** | **Vector Molar Mass (kg/mol)** | **DNA Molar Mass (kg/mol)** | **Asymmetry** | **Tailing Factor** | **Resolution to Dimer Peak** |
| 1 | 1.16E+13 | 1.06E+13 | 5.01E+03 | 1203.3 | 1.69 | 1.44 | 2.0 |
| 2 | 1.12E+13 | 1.01E+13 | 5.09E+03 | 1213.0 | 1.69 | 1.44 | 2.0 |
| 3 | 1.16E+13 | 1.06E+13 | 5.10E+03 | 1224.4 | 1.68 | 1.43 | 2.0 |
| 4 | 1.13E+13 | 1.02E+13 | 5.00E+03 | 1197.9 | 1.69 | 1.44 | 2.0 |
| 5 | 1.11E+13 | 1.00E+13 | 5.01E+03 | 1195.5 | 1.69 | 1.44 | 2.0 |
| 6 | 1.12E+13 | 1.02E+13 | 5.04E+03 | 1209.8 | 1.68 | 1.43 | 2.0 |
| 7 | 1.14E+13 | 1.03E+13 | 4.96E+03 | 1189.6 | 1.69 | 1.44 | 2.0 |
| 8 | 1.12E+13 | 1.02E+13 | 4.97E+03 | 1194.3 | 1.69 | 1.44 | 2.0 |
| 9 | 1.12E+13 | 1.02E+13 | 5.04E+03 | 1212.1 | 1.68 | 1.43 | 2.0 |
| 10 | 1.11E+13 | 1.01E+13 | 4.96E+03 | 1192.4 | 1.68 | 1.43 | 2.0 |
| **Dilution Corrected Mean** | **5.64E+13** | **5.13E+13** | **5.02E+03** | **1203.2** | **1.68** | **1.43** | **2.0** |
| **%CV** | **0%** | **0%** | **1%** | **1%** |  |  |  |
| **SRT 1000Å** | | | | | | | |
| **Replicate** | **CP/mL** | **VG/mL** | **Vector Molar Mass (kg/mol)** | **DNA Molar Mass (kg/mol)** | **Asymmetry** | **Tailing Factor** | **Resolution to Dimer Peak** |
| 1 | 1.14E+13 | 1.03E+13 | 5.05E+03 | 1210.5 | 1.51 | 1.30 | 2.6 |
| 2 | 1.15E+13 | 1.04E+13 | 5.06E+03 | 1211.8 | 1.51 | 1.30 | 2.6 |
| 3 | 1.13E+13 | 1.03E+13 | 5.06E+03 | 1210.1 | 1.51 | 1.30 | 2.6 |
| 4 | 1.15E+13 | 1.04E+13 | 5.08E+03 | 1216.2 | 1.51 | 1.30 | 2.6 |
| 5 | 1.15E+13 | 1.05E+13 | 5.06E+03 | 1212.4 | 1.51 | 1.30 | 2.6 |
| 6 | 1.12E+13 | 1.02E+13 | 5.06E+03 | 1212.4 | 1.51 | 1.30 | 2.6 |
| 7 | 1.13E+13 | 1.03E+13 | 5.04E+03 | 1204.8 | 1.51 | 1.30 | 2.6 |
| 8 | 1.15E+13 | 1.04E+13 | 5.06E+03 | 1211.7 | 1.51 | 1.30 | 2.6 |
| 9 | 1.12E+13 | 1.01E+13 | 5.02E+03 | 1200.8 | 1.52 | 1.31 | 2.6 |
| 10 | 1.11E+13 | 1.00E+13 | 5.04E+03 | 1205.1 | 1.51 | 1.30 | 2.6 |
| **Dilution Corrected Mean** | **5.67E+13** | **5.15E+13** | **5.05E+03** | **1209.6** | **1.51** | **1.30** | **2.6** |
| **%CV** | **0%** | **0%** | **0%** | **0%** |  |  |  |
