# Supplementary material for: Application of Size Exclusion Chromatography with Multiangle Light Scattering in the Analytical Development of a Preclinical Stage Gene Therapy Program
Source: Hum Gene Ther. 2023 Apr 17;34(7-8):325–38. doi: 10.1089/hum.2022.218 (PMC10125404; doi:10.1089/hum.2022.218)
Supplement: Supplemental data [file Supp_TableS2.docx]

**Supplemental Data Table 2** – Capsid ELISA and ddPCR Titer for Process Confirmation Material

|  | **Capsid ELISA** | **ddPCR Titer** |
| --- | --- | --- |
| **Tube** | **CP/mL** | **VG/mL** |
| 1 | 5.76E+13 | 4.34E+13 |
| 2 | 5.88E+13 | 4.44E+13 |
| 3 | 6.04E+13 | 4.54E+13 |
| 4 | 6.19E+13 | 4.54E+13 |
| 5 | 6.07E+13 | 4.53E+13 |
| 6 | 6.05E+13 | 4.18E+13 |
| 7 | 5.67E+13 | 4.23E+13 |
| **Mean Value** | **5.95E+13** | **4.40E+13** |
| **%CV** | **3%** | **3%** |
