# Supplementary material for: Application of Size Exclusion Chromatography with Multiangle Light Scattering in the Analytical Development of a Preclinical Stage Gene Therapy Program
Source: Hum Gene Ther. 2023 Apr 17;34(7-8):325–38. doi: 10.1089/hum.2022.218 (PMC10125404; doi:10.1089/hum.2022.218)
Supplement: Supplemental data [file Supp_TableS3.docx]

**Supplemental Data Table 3** – Purity Assessment of Empty Capsids and Non-GLP Vector by SDS-CE

| **Sample** | **Mean Migration Times (%CV)** | | | | **Mean Corrected Area (%CV)** | | |
| --- | --- | --- | --- | --- | --- | --- | --- |
|  | **VP3^a^** | **VP3** | **VP2** | **VP1** | **VP3^a^ + VP3** | **VP2** | **VP1** |
| Empty Capsids | 29.9 (0%) | 31.2 (0%) | 32.5 (0%) | 34.2 (0%) | 3471.0 (6%) | 525.2 (8%) | 289.0 (9%) |
| STRX-330 non-GLP material | 30.6 (2%) | 31.7 (2%) | 33.1 (3%) | 34.9 (2%) | 2503 (4%) | 347.3 (8%) | 269.0 (10%) |

^a^ truncated VP3 peak
