# Supplementary material for: Application of Size Exclusion Chromatography with Multiangle Light Scattering in the Analytical Development of a Preclinical Stage Gene Therapy Program
Source: Hum Gene Ther. 2023 Apr 17;34(7-8):325–38. doi: 10.1089/hum.2022.218 (PMC10125404; doi:10.1089/hum.2022.218)
Supplement: Supplemental data [file Supp_TableS4.docx]

**Supplemental Data Table 4** – Comparison of Full-to-Empty Results Across Methods

| **Full and Empty Results^a^** | | | | | |
| --- | --- | --- | --- | --- | --- |
|  | **SV-AUC** | **Cryo-EM** | **SEC-MALS** | **ddPCR/ELISA** | **Stunner** |
| Sample 1 (Full) | 0.899 | 0.992 | 0.916 | 0.900 | 0.744 |
| Sample 2 | 0.777 | 0.698 | 0.729 | 0.632 | 0.528 |
| Sample 3 | 0.611 | 0.473 | 0.531 | 0.379 | 0.369 |
| Sample 4 | 0.431 | 0.306 | 0.412 | 0.306 | 0.254 |
| Sample 5 | 0.311 | 0.204 | 0.256 | 0.219 | 0.154 |
| Sample 6 (Empty) | 0.004 | 0.010 | 0.001 | 0.003 | ND^b^ |

^a^ Data shown are the full capsid to total capsid ratio determined with each method.

^b^ ND, not detected
