# Supplementary material for: Application of Size Exclusion Chromatography with Multiangle Light Scattering in the Analytical Development of a Preclinical Stage Gene Therapy Program
Source: Hum Gene Ther. 2023 Apr 17;34(7-8):325–38. doi: 10.1089/hum.2022.218 (PMC10125404; doi:10.1089/hum.2022.218)
Supplement: Supplementary file 5 [file Supp_FigS1.docx]

STRX-330 

non-GLP material

System Peaks

Empty Capsids

System Peaks

Supplemental Fig.1

Peak Resolution ≥ 2.0

Asymmetry ≤ 1.5
