# Supplementary material for: Application of Size Exclusion Chromatography with Multiangle Light Scattering in the Analytical Development of a Preclinical Stage Gene Therapy Program
Source: Hum Gene Ther. 2023 Apr 17;34(7-8):325–38. doi: 10.1089/hum.2022.218 (PMC10125404; doi:10.1089/hum.2022.218)
Supplement: Supplementary file 6 [file Supp_FigS2.docx]

Sample 1 (full)

Sample 2

Supplemental Fig.2

Sample 4

Sample 3

Supplemental Fig.2

Sample 6 (empty)

Sample 5

Supplemental Fig.2
