# Supplementary material for: Application of Size Exclusion Chromatography with Multiangle Light Scattering in the Analytical Development of a Preclinical Stage Gene Therapy Program
Source: Hum Gene Ther. 2023 Apr 17;34(7-8):325–38. doi: 10.1089/hum.2022.218 (PMC10125404; doi:10.1089/hum.2022.218)
Supplement: Supplementary file 7 [file Supp_FigS3.docx]

Supplemental Fig.3A

Sample 1 (full) 99.2% full

Sample 2 69.8% full

Sample 3 47.3% full

Sample 4 30.6% full

Supplemental Fig.3A

Sample 5 20.4% full

Sample 6 (empty)

Supplemental Fig.3A
